# Fundamental insight into critical phenomena in condensation growth of nanoparticles in a flame

**DOI:** 10.1038/s41598-022-20210-x

**Published:** 2022-09-20

**Authors:** Igor Altman, Elena Fomenko, Igor E. Agranovski

**Affiliations:** 1 Combustion Sciences and Propulsion Research Branch, Naval Air Warfare Center Weapons Division, 1 Administration Circle, China Lake, CA 93555 USA; 2 School of Engineering and Built Environment, Griffith University, Nathan, QLD 4111 Australia

**Keywords:** Nanoparticles, Nanoparticles

## Abstract

The paper deals with the gas-phase formation of nanoparticles that is a fundamental process responsible for the condensed matter in the Universe, which also attracts attention due to its involvement in the particle synthesis for various nanotechnology applications. Previously reported results on MgO nano-oxides formed by Mg combustion showed a unique phenomenon coined “the condensation stagnation” that is the occurrence of critical clusters with suppressed growth. Here we focus on the effect of an external ionizer on this condensation growth stagnation. We show that the condensation stagnation occurring in the Mg particle flame subjected to a positive ion flux is similar to that in the unaffected flame. In contrast, applying negative charging significantly influences the state of stagnation of the system, i.e., no critical clusters are observed in the products sampled from the flame. The discovered critical behavior of the state of stagnation is explained in terms of the heat transfer between the condensed MgO nanoparticles and the surrounding gas, which efficiency depends on the sign of the nanoparticle charge. This dependence of the heat transfer efficiency on the nanoparticle charge is a new fundamental effect that should become the basis for accurate modeling in two-phase high-temperature systems.

## Introduction

Being a fundamental process responsible for creating the condensed matter in the Universe^1^, the gas-phase formation of nanoparticles^2–4^ is also a key issue in many applications. For research purposes, the process can be realized in metal-containing flames^5,6^. The formed gaseous metal suboxides further condense generating nano-oxides^7^. It is also the basis of flame synthesis of various nanomaterials.

The distinctive peculiarity of the process is a relatively high energy release corresponding to the transition from a gaseous to a condensed (liquid or solid) state that is on the order of 5 eV per condensing molecule^8^. Due to that high value of the released energy, its dissipation, which is an essential condition of the nanoparticle growth, is a complex process that can lead to critical phenomena in the evolution of generated particulate^9,10^. The energy dissipation mechanisms have not be paid enough attention in the current models describing nanoparticle formation. Historically, it is related to the apparently obvious assumption on the thermal equilibrium between generated nanoparticles and the bath gas, which implies the efficient heat transfer on the particle-gas interface^11^. On the contrary, an inefficient heat transfer can lead to a critical nanoparticle evolution during formation that is caused by an impossibility to dissipate the condensation energy. Thus, the question on heat transfer on the nanoparticle-gas interface is a key issue for fundamental understanding of the particulate generation from a gas.

It should be emphasized that the Mg flame is probably an optimal system to study the gas-phase formation of nano-oxides. The MgO nanoparticles formed as a result of combustion have the perfect cubical shape that evidences the solid state of material during the growth^12,13^. It allows for ruling out the formation mechanisms alternative to condensation such as coalescence, which is historically considered as the leading growth process^11^.

In our previous work, analyzing products sampled from a single Mg particle flame we discovered and addressed a unique phenomenon coined “the condensation stagnation”^13^. Two groups of particles occur in combustion products, specifically, mature cubes of sizes distributed around 30–40 nm, and shapeless particles of about 6–7 nm typical size. We call those shapeless particles “critical clusters”. There is the distinctive gap between the particle size distributions, i.e., there are no particles in the size range between ~ 10 nm and ~ 15 nm. The phenomenon is based on peculiarities of the heat transfer between the MgO nanoparticle and bath gas. At a low energy accommodation coefficient (EAC)^14,15^ describing efficiency of the heat transfer between the condensed body and the gas in the free-molecular regime, the nanoparticle becomes thermally isolated from the gas. Then, overheating of a critical cluster upon an arrival of a single oxide molecule leads to a loss of saturation conditions required to sustain condensation^13^. It is this overheating that does not allow for the condensation growth of critical cluster resulting in their stagnation.

Based on understanding of its mechanism that is related to heat transfer, the stagnation can be controlled by varying the EAC. In particular, a significant increase of the EAC should lead to the stagnation disappearance due to the loss of stagnation conditions related to the thermal isolation of critical clusters. The recent author’s results on the EAC comprehension^15^ provide a guidance on the sought stagnation control. The nanoparticle charging can be a mechanism of the stagnation switch.

In this paper we focus on the effect of external charging on the condensation stagnation and demonstrate the phenomenon control depending on the sign of charging. In particular, it is shown that the stagnation disappears under negative charging that corresponds to the theory prediction on the EAC increase^15^, and, therefore, to the loss of the thermal isolation at these conditions. This demonstration of the condensation stagnation control by varying the EAC is a key step in creating the accurate approach to the description of the gas-phase nanoparticle formation.

Besides the fundamental importance for comprehending nanoparticle condensation growth in high-temperature systems, the discovered stagnation-related phenomena have practical implications. A possibility to affect that condensation stagnation would undoubtedly allow for controlling the final properties of generated particulate such as the PSD, which is beneficial for a fine tailoring of nanoparticle morphology in the flame synthesis^2,3^. Also, the stagnation reduction would result in the condensation enhancement. That enhanced condensation is, in particular, preferred in propulsion applications in order to shift the process from the nozzle toward the combustor, which leads to increase of the condensation energy contribution to the system thrust^16^.

## Results and discussion

The purpose of the experiments was to obtain a snapshot of the flame-generated particulates that could be further examined. In order to get reliable data, the traditional approach to the nanoparticle sampling using a thermophoretic probe^17^ is not applicable. The problem is related to the flame disturbance and the relatively long residence time of the probe during this type of sampling. It could be solved by using an alternative sampling procedure that allows for a short transmission electron microscope (TEM) grid residence time in the flame. If this were the case, the TEM images would represent the actual particle morphology, which is not affected by the above-mentioned drawbacks. Our previously developed experimental setup was proposed for instant deposit of particles on a TEM grid for following visualization on high resolution TEM^12,13^.

**Figure 1:**
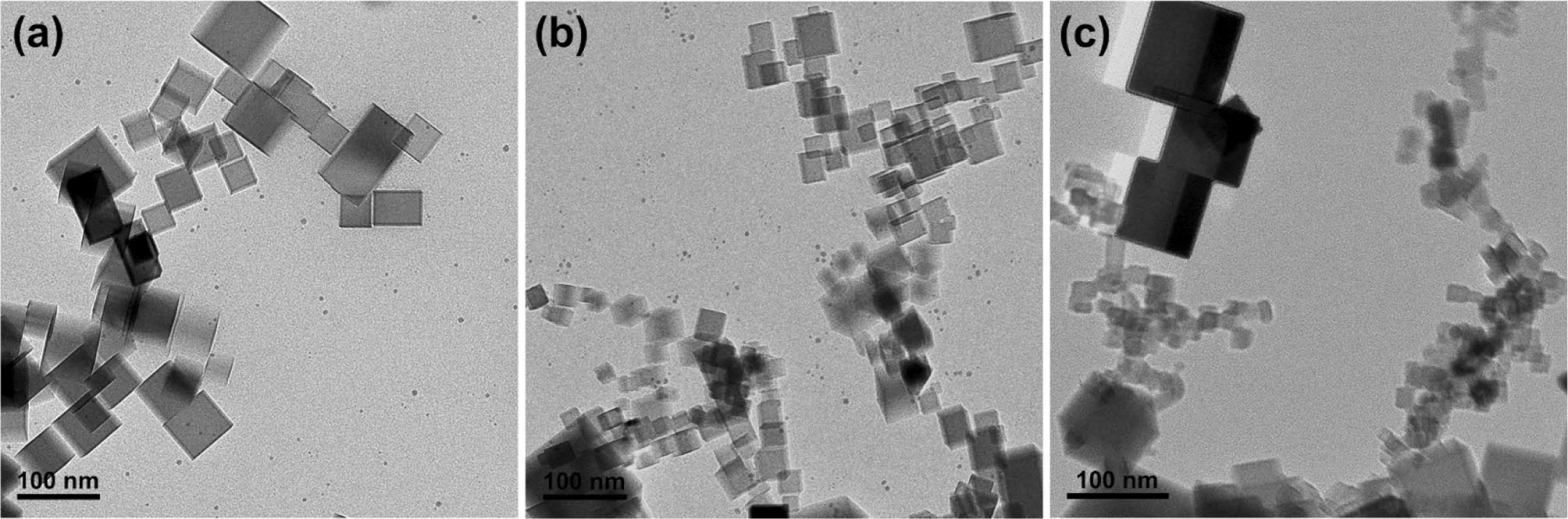
The typical TEM images of samples collected from (**a**) neutral, (**b**) positive, and (**c**) negative Mg particle flames. Sub-ten nanometer-size clusters (small dots) are seen together with mature cubical particles in neutral and positive flames. Those clusters disappear in the negative flame followed by an appearance of the 10–15 nm size particles.

**Figure 2:**
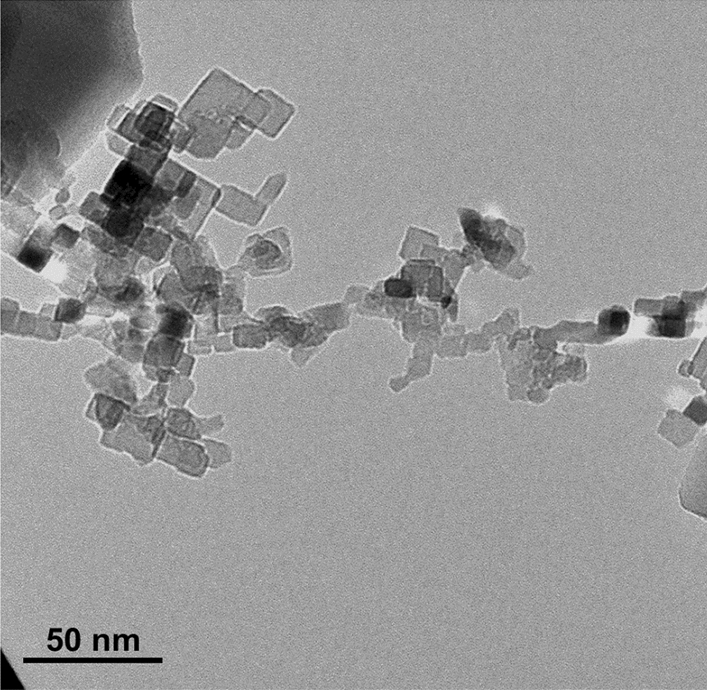
TEM showing the 10–15 nm size particles that appear only in the negative Mg flame.

The typical TEM images of samplescollected from a neutral Mg flame (no external charging was applied), as well as flames exposed to “positive” or “negative” external charging (a home-made ionizer^18^ was utilized) are presented in Fig. 1. In addition, some selective area of the sample collected from the Mg flame exposed to negative ions is shown in Fig. 2.

**Figure 3:**
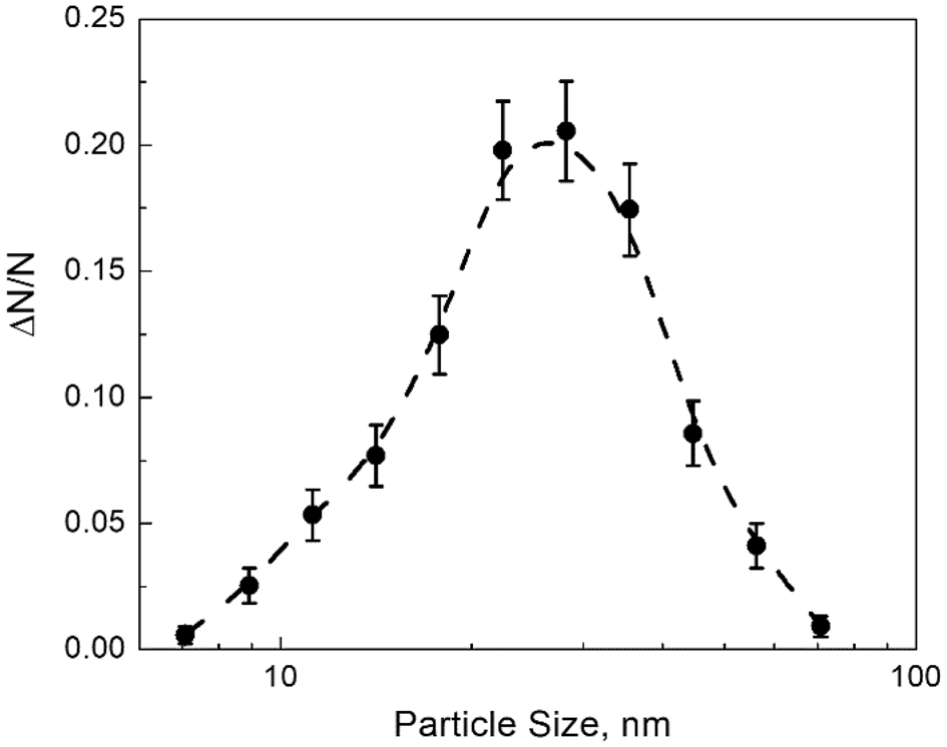
Size distribution of MgO nanoparticles collected from the negative flame. Error bars correspond to the possible errors in the number of particles contributing to given size intervals. The dashed line is a guide to the eye. The noticeable amount of the 10–15 nm size particles is observed.

The PSD of the particulates collected from the negative flame is presented in Fig. 3. About 500 particles were observed. The error bars shown in Fig. 3 correspond to the possible errors in the number of particles contributing to the given size intervals, i.e., δ(Δ*N*)≡(Δ*N*)^1/2^.

The following observations can be summarized for further analysis:

- Besides mature cubical particles of tens of nanometers size, sub-ten nanometer-size clusters are present in the neutral and positive flames. Those clusters do not exist in the negative flame;
- The 10–15 nm size particles are present in the negative flame instead of sub-ten nanometer-size clusters that occur in the neutral and positive flames.

The occurrence of shapeless sub-ten nanometer-size clusters in the neutral Mg particle flame has previously been discovered and addressed^13^. The phenomenon was coined “stagnation of condensation growth”. Its mechanism is based on the thermal isolation of clusters from the bath gas. That thermal isolation is possible at a low EAC, which is an efficiency of conductive heat transfer on the interface, i.e., the ratio of the energy transferred upon a single molecule collision to that at the full accommodation. Note that in literature two terms are used for the same, namely, the energy accommodation coefficient and the thermal accommodation coefficient^19^. In our opinion, since it deals with energy, the term “energy accommodation coefficient” is more accurate to describe the process. Under thermal isolation of clusters with the bath gas, the arrival of a single condensing oxide molecule leads to the cluster overheating. At the elevated cluster temperature, the surrounding oxide vapor becomes non-saturated being supersaturated before that single condensation event. As a result, the condensation growth of clusters is impossible and the stagnation occurs.

Based on the previous knowledge, it is natural to assume that the disappearance of sub-ten nanometer-size clusters in the negative flame originates from the loss of conditions that sustain their occurrence in the neutral flame. That loss of conditions can be possible if the EAC increases reducing the thermal isolation of clusters from the bath gas. In its turn, this enables a single condensation event without the significant cluster overheating that would have led to the loss of supersaturation. Upon the loss of conditions sustaining the stagnation, clusters can further grow via condensation, which results in the appearance of particles of 10–15 nm size. Those particles are seen in the negative flame only. Then, it is natural to assume that they originate from grown clusters, which stagnate in the neutral and positive flames. It should be noted that the size of these 10–15 nm particles being grown from stagnating clusters fills the gap in the PSD resulting from the condensation stagnation in the neutral flame (see Fig. 3^13^); this Figure is also reproduced in Methods section). This explanation requires fundamental understanding of reasons why and how the EAC increases in the negative flame and is likely unaffected in the positive flame.

Our explanation is based on the recent EAC concept^15^. Two mechanisms contribute to the EAC, specifically, the phonon-related channel that occurs in any material, and the electron-related channel, which is possible only in relatively-high electrically conductive materials. The phonon-related component of the EAC is vanishing at high temperatures^14^, and it is the charge independent. The electron-related component occurring only in metal-like materials is sensitive to the particle charge^15^. At the positive nanoparticle charge, the Coulomb blockage prevents electron from reaching the particle surface that suppresses the electron-related component of the EAC. At the negative nanoparticle charge, the effect is absent resulting in the high EAC.

A relatively high energy is released during nano-oxide condensation from the gas phase, which in the case of MgO:

1 $$\mathrm{MgO}\left(\mathrm{gas}\right)=\mathrm{MgO}\left(\mathrm{solid}\right),$$

is about 660 kJ/mol^8^ that is the equivalent of 6.8 eV per condensing molecule resulting in unusual nanoparticle properties revealed in our previous work. In particular, it was demonstrated that nano-oxide particles formed in the flame exhibit metal-like electrical conductivity^20^_._ The latter justifies a need to consider the electron-related component of the EAC along with the phonon-related component while describing the nano-oxide heat transfer with the bath gas. Note that within the flame nano-oxides acquire positive charge due to the thermionic emission of electrons caused by the high particle temperature. Then, the effective EAC, *α*, can be expressed as

2 $$\alpha ={\alpha }_{ph}+{\alpha }_{el},$$

where the charge independent phonon component, *α*_*ph*_, and the electron component, *α*_*el*,_ depends on the positive charge, *Z*, as^15^ ${\alpha }_{el}\left(Z\right)\propto {\mu }^{-Z}$. The factor *µ* that controls the electron component of the EAC $\mu \propto \mathrm{exp}\left(\frac{{k}_{e}{e}^{2}}{D{k}_{B}T}\right)$, with *k*_*e*_ and *k*_*B*_ being the electrostatic and Boltzmann constants, accordingly, and *e* being the elementary charge. Thus, the EAC depends on the acquired charge as

3 $$\alpha \left(Z\right)={\alpha }_{ph}+{\mu }^{-Z} ,$$

**Figure 4:**
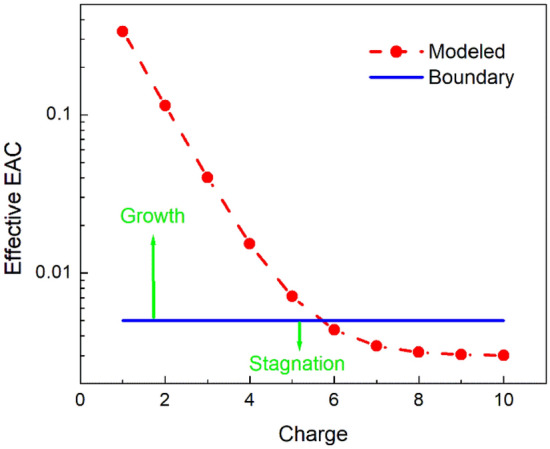
The effective EAC vs. the positive nanoparticle charge. The dashed line is a guide to the eye. The horizontal line is the boundary between areas of “condensation growth” and “condensation stagnation”.

Figure 4 illustrates the EAC as a function of the acquired particle charge given by Eq. (3) at the constant value *α*_*ph*_ = 0.003, and at *µ* = 3 that is estimated at the cluster size *D* ~ 6 nm and the particle temperature *T* ~ 2500 K. The boundary between the EAC areas where either the condensation growth (higher EAC) or the condensation stagnation (lower EAC) occurs is also plotted for convenience. For illustrative purposes the boundary is shown at *α* = 0.004. Following the Fig. 4 concept, the clusters with the charge *Z* ≥ 6 should stagnate.

Like other physical values, the particle charge magnitude also has some distribution. Then, one can estimate the effect of external negative charging on stagnation. It can be quantified as the ratio of the number of clusters that stagnate in the negative and neutral flames.

4 $$\eta \equiv \frac{{\Delta N_{ - } }}{{\Delta N_{0} }},$$

where Δ*N*_*-*_ and Δ*N*_*0*_ are the number of clusters that carry the charge providing the EAC value favorable for stagnation in the negative and neutral flames, correspondingly. Assuming that external charging reduces the particle charge by 1, one can conclude that if the clusters with the charge *Z* ≥ Z^*^ stagnate in the neutral flame then the clusters carrying the charge *Z* ≥ (Z^*^ + 1) before the ion exposure would stagnate in the negative flame. Thus,

5 $$\eta =\frac{{\sum }_{{Z}^{*}+1}^{\infty }N\left(Z\right)}{{\sum }_{{Z}^{*}}^{\infty }N\left(Z\right)} ,$$

with *N*(*Z*) being the function describing the particle charge distribution.

From Eq. (5), one can understand that *η* < 1, which is generally means the stagnation reduction. Using the Gaussian distribution of the particle charge $N\left(Z\right)\propto \mathrm{exp}\left[-{\frac{1}{2}\left(\frac{Z-\overline{Z}}{\sigma }\right)}^{2}\right]$ in Eq. (5) and choosing the average particle charge $\overline{Z }=4$ and the distribution dispersion *σ* = 1, at Z^*^ = 6 one can get *η* = 0.08. This low value of *η* can be interpreted as the stagnation suppression/disappearance corresponding to the above observations. Thus, this illustrative estimate supports a feasibility of the effect of external negative charging on the EAC, which is required to explain the experiment.

In order to analyze an ionizer capability to affect the charge balance of the flame, the ionizer current, ${I}_{ion}$, that is on the order of single microamperes should be compared with the effective flame current,${I}_{fl}$, which can be defined as the flame-associated charge crossing a horizontal plane per unit time due to natural convection. This flame current can be estimated as

6 $${I}_{fl}=\frac{{Q}_{fl}}{L}v$$

with *v* being the convection velocity and *L* being the flame diameter.

The flame-associated charge is related to the charge number density, *n*, and the flame volume occupied, *V* ≡ *πL*^3^/6, as

7 $${Q}_{fl}=enV=\frac{\pi en{L}^{3}}{6}.$$

Then,

8 $${I}_{fl}=\frac{\pi en{L}^{2}}{6}v.$$

At the typical flame charge number density *n* = 10^18^ m^−3^, and *L* = 6 mm (twice the burning particle size) and *v* = 40 cm/s^21^, we obtain *I*_*fl*_ = 1.2 µA. Thus, the ionizer current is comparable with the flame current, which means that the ionizer is capable of affecting the flame charge balance.

## Concluding remarks

The major finding of the current work is the demonstration of a disappearance of the condensation growth stagnation in the Mg particle flame exposed to external negative charging. The phenomenon can be explained based on the strong variation of the energy accommodation coefficient depending on the nanoparticle charge. This EAC control is a fundamental result that needs a close attention while modeling two-phase systems at high temperatures. The effect of the EAC variation is expected to be strong in metal-containing flames due to a high energy release during metal combustion that sustains high temperatures within the system. The thermionic emission of electrons from nano-oxides formed in the system is an internal mechanism of the EAC change. The EAC can be also affected by external charging. The condensation stagnation phenomenon and the possibility to control it can have important implications in high-temperature particulate-generating systems when a fine tailoring of particle morphology is essential.

It should be emphasized that the relatively high energy release during nano-oxide condensation, which is the reason for phenomena considered in this work, is general for metal-containing flames. Furthermore, the condensation energy dissipation by condense-luminescence^22^ that controls the rate of nano-oxide growth is also of general nature. Then, the results could be extrapolated from Mg to other metals. A Mg flame studied in this paper is just an optimal system allowing for separating effects relevant to condensation, which is possible due to the perfect cubical shape of MgO nanoparticles.

## Methods

The goal of experiments was to obtain a snapshot of the flame-generated particulate that could be further examined. The common approach is to deposit particles on a transmission electron microscope (TEM) grid, and then, to analyze it with TEM. In order to get reliable data, the traditional approach to nanoparticle sampling using a thermophoretic probe^17^ is not applicable. The issue is related to the flame disturbance and the relatively long residence time of the probe during sampling. The problem can be overcome utilizing the sampling that allows for a short TEM grid residence time. If the residence time of the grid within the flame is short enough, TEM images would represent the actual particulate morphology that is not affected by the flame disturbance due to the sampling.

**Figure 5:**
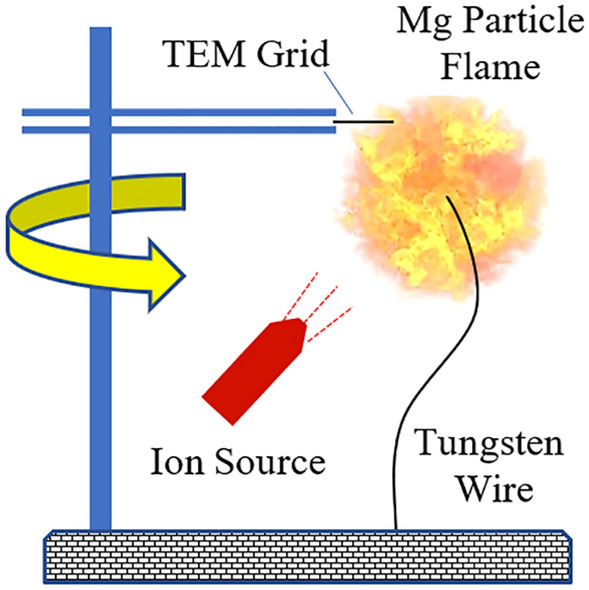
The sketch of the experimental set-up utilized for collection of MgO nanoparticles from a single Mg particle flame.

The experimental set-up is sketched in Fig. 5 below. The flame of a single Mg particle was studied for purposes of the combustion product analysis. A magnesium cube of ~ 3-mm side supported by a 0.25-mm tungsten wire allowed for a steady flame with the typical burn time on the order of 10 s. The magnesium cube had a blind hole of 0.5-mm diameter required to secure it on the wire. The particle was ignited by a portable propane-air diffusion flame that was immediately removed after the particle ignition. A vertical rod with attached a tweezers-type support holding the TEM grid, which plane was horizontal, was utilized for the product sampling. The same grid position relative to the Mg particle was manually verified before each run. The sampling was realized by the quick rod rotation around its vertical axis, when MgO nanoparticles were deposited on the TEM grid during the short residence time of the grid within the zone of particle generation. That residence time of the grid in the generation zone was about 1 ms^12^ in all experiments. The sampling was performed in about 2 s after ignition to ensure flame stabilization. MgO nanoparticles were sampled from the unaffected flame and from the flame, to which the unipolar emission of ions was applied. Both the negative and positive ion emission were utilized and generated by a home-made ionizer capable of producing both positive and negative ions^18^. The distance from the ionizer to the burning particle was about 5 cm. The whole set-up was placed on a metal plate that was electrically grounded. Five sampling runs were performed for each charging scenario to confirm repeatability of the nanoparticle morphology peculiarities.

The MgO nanoparticles deposited on the grids were analyzed with TEM (JEM-1400, JEOL Ltd., Tokyo, Japan). Obtained TEM images were visually examined. The specimen collected from the negative flame was processed to obtain the PSD. That PSD illustrates the nanoparticle morphology difference concluded from TEM images with naked eyes.

**Figure 6:**
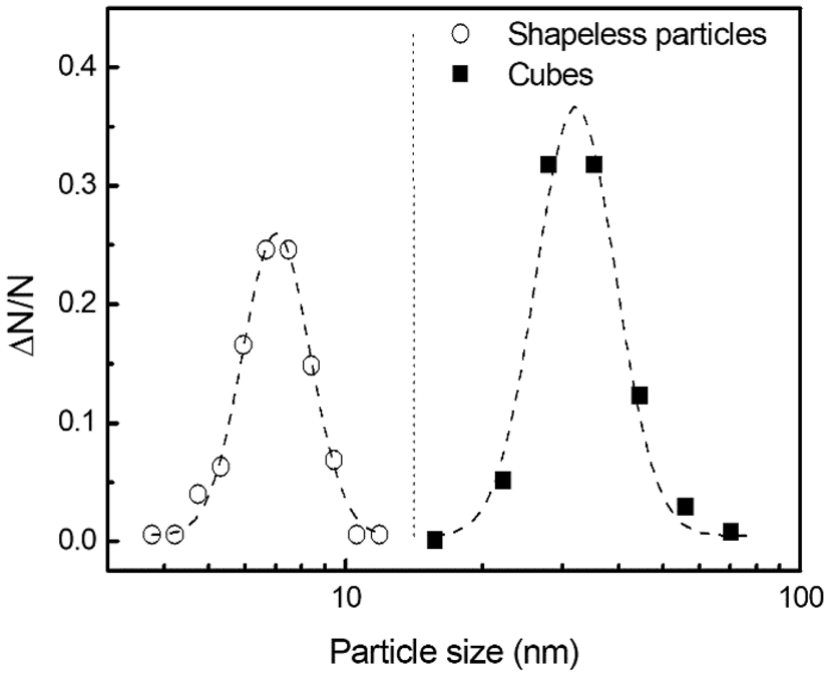
Size distribution of MgO particles collected from the neutral flame (adapted from^13^). The gap between PSDs is clearly seen in the size range of 10–15 nm.

In order to obtain the PSD, logarithms of nanoparticles sizes measured from TEM images were combined in a single-column database and further processed using the built-in Origin “Frequency Count” procedure to separate data among bins. The bin size of 0.1 (logarithmic scale) was chosen in order to get 10 size intervals contributing to the PSD. A number of particles in a given bin was divided by the total number of particles, which is reported as Δ*N*/*N* in the PSD Figure. The PSD of the particulate collected from the negative flame was compared with the PSD reported for the neutral flame (see Fig. 3^13^) that is also shown in Fig. 6.

## Data Availability

All data that support the plot within this paper and other findings of this study are available from the corresponding author upon reasonable request.
